# Biological Significance of Probiotic Microorganisms from Kefir and Kombucha: A Review

**DOI:** 10.3390/microorganisms12061127

**Published:** 2024-05-31

**Authors:** Talita Andrade da Anunciação, Juan Diego Silva Guedes, Pedro Paulo Lordelo Guimarães Tavares, Fernando Elias de Melo Borges, Danton Diego Ferreira, Jorge Alberto Vieira Costa, Marcelo Andrés Umsza-Guez, Karina Teixeira Magalhães-Guedes

**Affiliations:** 1 Post-Graduate Program in Food Science, Bromatological Analysis Department, Pharmacy Faculty, Federal University of Bahia (UFBA), Barão of Jeremoabo Street, s/n, Ondina, Salvador 40171-970, BA, Brazil; tali.anunciacao@hotmail.com (T.A.d.A.); pp.lordelo@gmail.com (P.P.L.G.T.); 2 Post-Graduate Program in Industrial Engineering, Polytechnic School, Federal University of Bahia (UFBA), Street Professor Aristídes Novis, 02, Federação, Salvador 40210-630, BA, Brazil; juandiegoguedes@yahoo.com.br; 3 Post-Graduate Program in Systems Engineering and Automation, Department of Engineering, Federal University of Lavras (UFLA), University Campus, Lavras 37000-200, MG, Brazil; fernandoelias.mb@gmail.com (F.E.d.M.B.); danton@ufla.br (D.D.F.); 4 Laboratory of Biochemical Engineering, College of Chemistry and Food Engineering, Federal University of Rio Grande (FURG), Rio Grande 474-96203-900, RS, Brazil; jorgealbertovc@gmail.com

**Keywords:** gut, kefir, immune system, probiotic microorganisms, kombucha

## Abstract

(1) Background: The human microbiota is essential for maintaining a healthy body. The gut microbiota plays a protective role against pathogenic bacteria. Probiotics are live microorganisms capable of preventing and controlling gastrointestinal and balancing the immune system. They also aid in better nutrients and vitamins absorption. Examples of natural probiotic cultures are kefir and kombucha. (2) Methods: Therefore, the aim of this review was to address the beneficial properties of probiotic kefir and kombucha using a Boxplot analysis to search for scientific data in the online literature up to January 2024: (Latin American and Caribbean Health Sciences (LILACS), PubMed, Medical Literature Analysis (MED-LINE), Science Direct, Google Scholar/Google Academic, Bioline Inter-national and Springer Link). Boxplots showed the summary of a set of data “Index Terms—Keywords” on kefir and kombucha in three languages (English, Portuguese and Spanish). (3) Results: Google Scholar was the database with the highest number of articles found, when the search for the keywords used in the study (containing ~4 × 10^6^–~4 million articles available). This was Followed by the Science Direct database, containing ~3 × 10^6^–~3 million articles available, and the BVS databases—Biblioteca Virtual de Saúde (Virtual Health Library) e Lilacs, both containing a value of ~2 × 10^6^–~2 million articles available. The databases containing the smallest number of articles found were Nutrients and Medline, both containing a value of ≤0.1 × 10^6^–≤100 thousand articles. (4) Conclusions: Scientific studies indicate that kefir and kombucha certainly contain various functional properties, such as antimicrobial, antitumor, anticarcinogenic and immunomodulatory activity, in addition to having a microbiological composition of probiotic bacteria and yeasts. Kefir and kombucha represent key opportunities in the food and clinic/medical fields.

## 1. Introduction

In recent decades, dietary habits and lifestyle have undergone several changes, leading to an overload on the body’s systems, such as the consumption of processed foods and fast-food, and sedentary behavior. This scenario has prompted people to seek alternatives to improve their health and nutrition [1,2,3,4]. Functional clinical nutrition is a contemporary approach to promoting the science of nutrition, aiming to assess the interaction of the body with food. This is necessary to adequately nourish the body in terms of quantity and quality, providing all the essential nutrients for proper functioning. This is also necessary to ensure good digestion and adequate absorption of ingested nutrients [4,5,6,7].

In addition to a healthy diet, the human microbiota is essential to maintain a balanced body. The human microbiota, especially the gut microbiota, plays a protective role against pathogenic bacteria [3,8].

Dysbiosis is an irregularity in the gut microbial population. This can lead to the development of a chronic inflammatory state. Dysbiosis causes symptoms such as gas, diarrhea, or constipation, and it is also related to cardiovascular diseases, metabolic syndromes, central nervous system disorder and immunological alterations [3,8]. Thus, probiotic foods/beverages are seen as improving both intestinal microbiota and human well-being [3,8].

Probiotics are live microorganisms capable of preventing and controlling gastro-intestinal issues and balancing the immune system [9,10,11,12,13,14]. They also combat allergies, aid in de-toxification, and regulate the body for better nutrient and vitamin absorption [9,10,11,12]. Currently, there is a variety of microorganisms used to produce different probiotic foods, such as lactic acid bacteria, acetic acid bacteria and yeasts [9,10,11,12,15,16,17,18,19,20,21,22,23,24,25,26,27,28,29,30,31,32,33,34,35]. However, this study demonstrates the benefits of natural probiotic cultures in kefir [15,16,17,18,19,20,21,22,23,24,25,26,27,28,29,30,31] and kombucha [32,33,34,35].

Kefir is a fermented beverage produced by the action of lactic acid bacteria, acetic acid bacteria and yeast, which coexist in symbiotic association in kefir grains [15,16,17,18,19,20,21,22]. The large number of probiotic microorganisms present in kefir, the bioactive compounds resulting from microbial metabolism, and the benefits associated with its regular consumption confer on kefir the status of a natural probiotic culture. Studies have shown that kefir and its metabolites have antimicrobial, antitumor, anticarcinogenic and immunomodulatory activity [23,24,25,26,27,28,29,30,31].

Kombucha is a food that has become popular within the current market trends regarding the search for healthier food [32,33,34,35]. Kombucha is an Asian, sweet, fermented beverage made from green tea and/or black tea (*Camellia sinensis*). Fermentation is caused by a symbiotic association of bacteria and yeast, forming a film called SCOBY (Symbiotic Culture of Bacteria and Yeasts) with a protein and fibrous constitution [32,33,34,35]. SCOBY can also be used as an inoculum for the formulation of fermented probiotic beverages, such as fruit kombucha. Several studies have reported beneficial properties of this fermented beverage, such as antimicrobial, anti-inflammatory, anticancer, antihypertensive, anti-diabetic and hepatoprotective activity [32,33,34,35].

Therefore, the aim of this review was to address the beneficial properties of probiotic kefir and kombucha using a Boxplot to search scientific data in the online literature. For this study, a search was carried out in online databases until January 2024: (Latin American and Caribbean Health Sciences (LILACS), PubMed, Medical Literature Analysis (MEDLINE), Science Direct, Google Scholar/Google Academic, Bioline International and Springer Link). Descriptive statistics were applied to analyze the data found. The descriptive statistics used in this study were via a Boxplot or Box-and-Whisker plot, which is a type of graph used in data intelligence analysis. Intelligent Boxplots visually display the distribution of numerical data and skewness, showing quartiles of data (or percentiles) and means. Boxplots provide a summary of a dataset, including the minimum score, first quartile (lower), median, third (upper) quartile, and maximum score for different “Index Terms—Keywords” [36,37].

## 2. Methods

For the elaboration of this study, a search was carried out in online databases until January 2024: (Latin American and Caribbean Health Sciences (LILACS), PubMed, Medical Literature Analysis (MEDLINE), Science Direct, Google Scholar/Google Academic, Bioline International and Springer Link). The “Index Terms” used for searches were developed in three languages (English, Portuguese and Spanish). English was used because it is the most common language in scientific articles worldwide. Portuguese is the authors’ native language. Spanish is the most frequently used language in South America, where Brazil is located, the authors’ resident country. Keywords in English = “probiotics microorganisms”, “kefir probiotic”, “symbiotic culture”, “kefiran”, “non-dairy probiotic beverage”, “functional beverage”, “kombucha probiotic”, “functional kombucha beverage” and “functional kefir beverage”. Keywords in Spanish = “microorganismos probióticos”, “kéfir probiótico”, “cultivo simbiótico”, “kefiran”, “bebida probiótica no láctea”, “bebida funcional”, “probiótico kombucha”, “bebida kombucha funcional” and “bebida de kéfir funcional”. Keyword in Portuguese = “micro-organismos probióticos”, “kefir probiótico”, “cultura simbiótica”, “kefiran”, “bebida probiótica não láctea”, “bebida funcional”, “kombucha probiótico”, “bebida funcional de kombucha” and “bebida funcional de kefir”.

Initially, the title/abstract was read, noting the year of publication, methodology used, and main results described. Subsequently, a Boxplot analysis of the data surveyed in the different studied databases was carried out. The data was grouped into 3 groups [36,37]:

1. Repository: this group has 9 clusters, each made up of the repository with its respective number of articles;
2. Language: this group has 3 clusters, each consisting of the idiomatic expression of the papers with the respective number of articles;
3. Keyword: this group has 9 clusters, each made up the keyword of the paper (regardless of the language of the article) with their respective number of articles.

After obtaining the groups, Boxplot analyses were performed. The Boxplot constructs a graphic visualization of the statistical distribution of a variable. A Boxplot is a standardized way of displaying data distribution based on a five number (“minimum”, first quartile (Q1), median, third quartile (Q3), and “maximum”). It can provide information about outliers and what their values are. It can also indicate whether data are symmetric, how tightly data are grouped, and if and how data are skewed.

The edges of the box show the 25th and 75th percentiles, respectively, by the lower and upper edges, and point out if the edges are labelled as outliers. The Boxplot analysis has setting visualization options, one of them being the “Notched Boxplot”, where the user can see the difference in the medians of the variables and the confidence of this difference. If there is no overlap in the notches, then there will be a difference between the medians with 95% confidence [36,37]. Subsequently, all articles were selected by title, abstract and full text, excluding those that did not address subjects related to the main theme of this review.

## 3. Results

### Boxplot Analysis

Application of “Boxplot analysis” for analysis of the data researched in this study (number of articles in relation to languages and databases) was performed as described in the section “Methodology”. The results of these analyzes are described and explained in this section.

**Figure 1:**
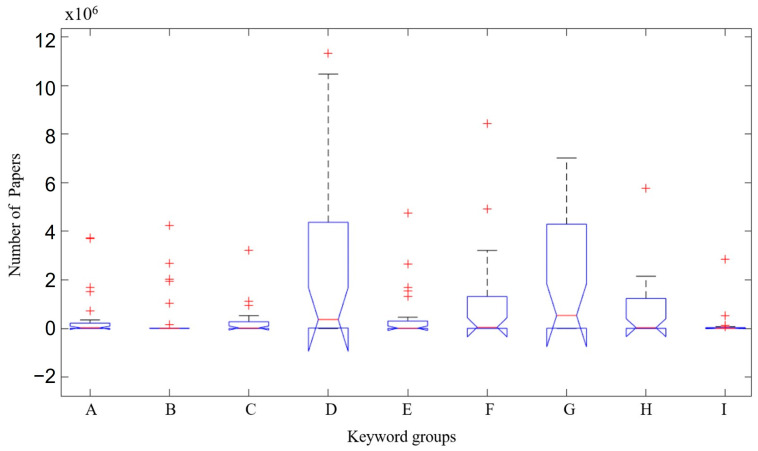
Distribution of number of papers by keywords searched. A group—Keyword in English (functional kombucha beverage); in Spanish (bebida kombucha funcional); in Portuguese (bebida functional de kombucha). B group—Keyword in English/Spanish/Portuguese (kefiran). C group—Keyword in English (functional beverage); in Spanish/Portuguese (bebida funcional). D group—Keyword in English (probiotics microorganisms); in Spanish (microorganismos probióticos); in Portuguese (micro-organismos probióticos). E group—Keyword in English (functional kefir beverage); in Spanish (bebida de kéfir functional); in Portuguese (bebida functional de kefir). F group—Keyword in English (kombucha probiotic); in Spanish (probiótico kombucha); in Portuguese (kombucha probiótico). G group—Keyword in English (kefir probiotic); in Spanish (probiótico kefir); in Portuguese (kefir probiótico). H group—Keyword in English (non-dairy probiotic beverage); in Spanish (bebida probiótica no láctea); in Portuguese (bebida probiótica não láctea). I group—Keyword in English (symbiotic culture); in Spanish (cultivo simbiótico); in Portuguese (cultura simbiótica). Red cross (+) are outliers (discrepant values in relation to the other analyzed values).

In Figure 1, the distribution of number of papers in the groups of keywords used is represented.

Group D—Keywords in English (probiotics microorganisms); in Spanish (microorganismos probióticos); in Portuguese (micro-organismos probióticos) and G group—Keywords in English (kefir probiotic); in Spanish (kéfir probiótico); in Portuguese (kefir probiótico) represent the largest number of articles found found(4 × 10^6^–~4 million articles) in the different searched databases (BVS—Biblioteca Virtual de Saúde (Virtual Health Library), Google Scholar, Springer, Science Direct, Nutrients, Scielo, Medline, Lilacs and PubMed). Followed by F group—Keywords in English (kombucha probiotic); in Spanish (probiótico kombucha); in Portuguese (kobucha probiótico) and H group—Keywords in English (non-dairy probiotic beverage); in Spanish (bebida probiótica no láctea); in Portuguese (bebida probiótica não láctea), containing both ~2 × 10^6^–~2 million articles. B group, Keywords in English/Spanish/Portuguese (kefiran), is the group with the smallest number of articles found at ≤0.1 × 10^6^–≤100 thousand articles.

The Boxplot shows that the terms “probiotics microorganisms—microorganismos probióticos—micro-organismos probióticos” and “kefir probiotic—kéfir probiótico—kéfir probiótico” are the most prevalent “Index Terms” in titles and keywords of scientific articles about kefir. These “Index Terms” characterize “Kefir” in relation to the scientific articles available in the three languages (English, Spanish and Portuguese) (Figure 1).

**Figure 2:**
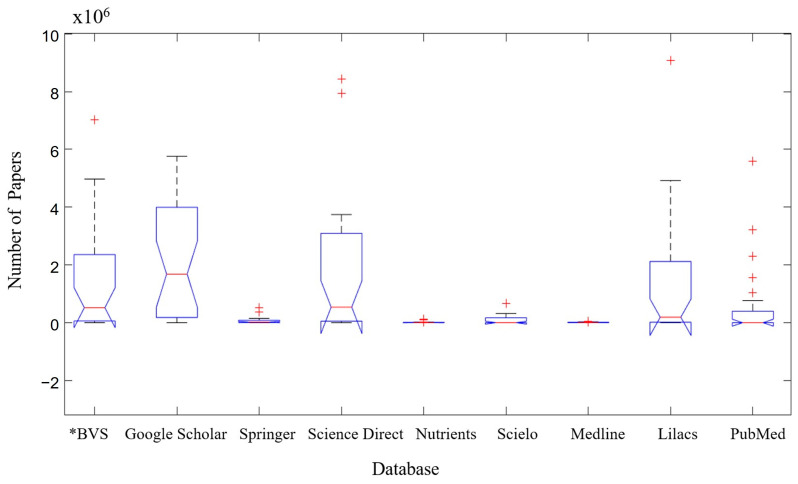
Number of papers by database searched. *BVS—Biblioteca Virtual de Saúde (Virtual Health Library). Red cross (+) are outliers (discrepant values in relation to the other analyzed values).

Figure 2 shows the distribution of number of papers by searched databases (BVS—Biblioteca Virtual de Saúde (Virtual Health Library), Google Scholar, Springer, Science Direct, Nutrients, Scielo, Medline, Lilacs and PubMed).

Google Scholar was the database with the highest number of articles found, when analyzing the search for keywords from all groups (A–I) in Figure 1, containing ~4 × 10^6^–~4 million articles available. This was followed by the Science Direct database, containing ~3 × 10^6^–~3 million articles available, and the BVS databases—Biblioteca Virtual de Saúde (Virtual Health Library) e Lilacs, both containing a value of ~2 × 10^6^–~2 million articles available. The databases with the lowest number of articles found were Nutrients and Medline, both containing a value of ≤0.1 × 10^6^–≤100 thousand articles. The notch of the google scholar database does not intersect the notch of the Springer, Nutrients, Scielo, Medline and Pubmed databases, so it can be said that the mean of the Google Scholar database is different from these with 95% certainty.

The Boxplot shows that the “Google Scholar” database presents the highest number of scientific articles in relation to all “Index Terms” used in the three languages (English, Spanish and Portuguese). The boxplot also considered the “Science Direct” database to be highly relevant for the search for scientific articles (Figure 2).

**Figure 3:**
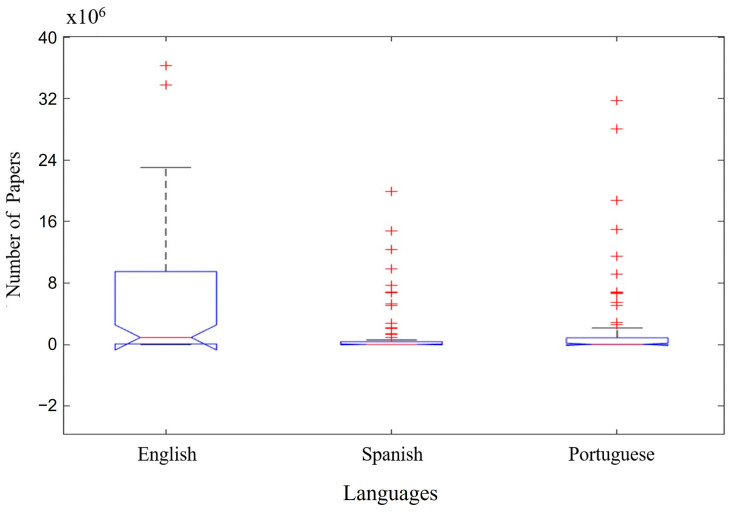
Distribution of number of papers by languages searched. Red cross (+) are outliers (discrepant values in relation to the other analyzed values).

Figure 3 shows the distribution of number of papers by languages searched (English, Spanish and Portuguese), considering all groups of keywords (A–I) in Figure 1 and the different databases searched in Figure 2 (BVS—Library Virtual Health Library, Google Scholar, Springer, Science Direct, Nutrients, Scielo, Medline, Lilacs and PubMed). The highest number of articles found was in the English language, adding up to ~8 × 10^6^–~8 million available articles. The second-highest number of articles found was in the Portuguese language, adding up to ~0.1 × 10^6^–~100 thousand articles available. The lowest number of articles found was in the Spanish language, adding a value of ≤0.05 × 10^6^–~50 thousand available articles.

The Boxplot shows that the English language is predominant compared to the three languages (English, Spanish and Portuguese). English is also predominant for all “Index Terms” and “Databases” researched in this study. This demonstrates that the English language is considered universal in the scientific community.

Considering the two databases with the highest number of articles found, Google Scholar, followed by Science Direct, in Table 1 and Table 2 can be seen the correlation between the quantitative analysis of scientific articles and the respective database analyzed. We can see the predominance of the English language in relation to the number of available articles (Figure 3).

Regarding the Google Scholar database (Table 1), the highest number of available articles was with the keyword “probiotics microorganisms” (993,000 articles available), followed by “kefir probiotic” (779,000 articles available) and “non-dairy probiotic beverage” (747,600 articles available). Regarding the Science Direct database (Table 2), the highest number of available articles was with the keyword “symbiotic culture” (31,111 articles available), followed by “probiotics microorganisms” (19,184 articles available) and “functional beverage” (6923 articles available). Boxplot analyzes show, the number of articles available in the English language predominant in both databases (Google Scholar and Science Direct). It is also possible to notice that the Science Direct database provides few articles in Spanish and no articles in Portuguese. This reinforces the general prevalence of scientific articles in the English language.

After the quantitative evaluation results from the Boxplot, articles were selected by title, abstract and full text, excluding those that did not address subjects related to the main focus of this review. A total of 83 articles were selected to discuss in this review. Among the 83 selected articles, 82 are in English and 1 in Portuguese.

## 4. Discussion

### 4.1. Historical Report for Kefir and Kombucha

The origin of kefir grains predates known records, but the first registers of similar beverages are from the Caucasus [15,16,17,18,19,20,21,22]. In ancient times, the Eastern nomadic shepherds discovered that the milk they carried in their leather purses during their travels would sometimes transform into a foamy beverage. The nomads called the beverage “kefir” and it is believed that the word originates from “keyif” which means “joy/pleasure” in Turkish [15,16,17,18,19,20,21,22]. There is also another legend that the Prophet Muhammad gave kefir grains to the people living in the mountains of the northern Caucasian, and they were then passed from generation to generation until the present day [15,16,17,18,19,20,21,22]. Kefir is also known as kefyr, kephir, kefer, kiaphur, knapon, kepi, and kippi in different countries all over the world [15,16,17,18,19,20,21,22].

Kombucha originates from East Asia. It was known in China for its energizing and detoxifying properties, where this beverage was named “Divine Che” [32,33,34,35]. After China, the popularity spread to Asia. There is a legend about the name: a physician named Kombu used the beverage (tea or “cha” in Japanese) to treat Emperor Inkyo, so the beverage was known as kobu’s tea, kombucha. From Japan, this beverage went to Russia (named “Tea Kvass”) and then spread throughout Europe and Africa. Nowadays, it is well known around the word [32,33,34,35].

### 4.2. Kefir: Microbiome and Preparation

Kefir is a fermented mildly effervescent beverage. The fermentation process for kefir is conducted by a natural microbiota present in the polysaccharide matrix of kefir [23,24,25,26,27,28,38,39]. Kefir grains are a symbiotic association composed mainly of yeasts, lactic acid bacteria and acetic acid bacteria. Kefir grains range in size from 0.5–3.5 cm in diameter, have an irregular shape, and a yellowish or whitish color [27,28,29,30,31,38,39].

**Figure 4:**
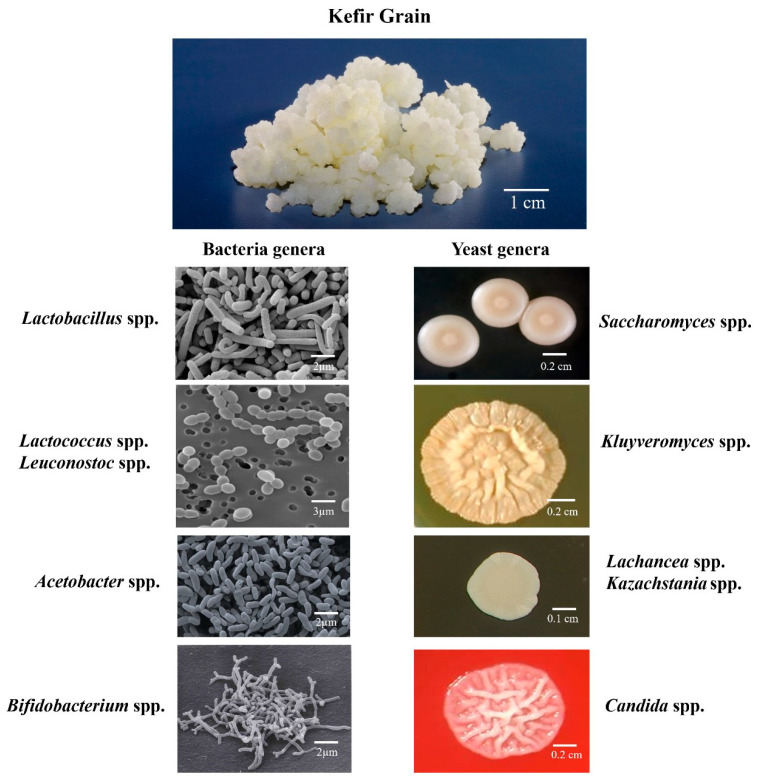
Bacteria/yeast genera present in kefir grains and kefir-based beverage. Original and unpublished figure (authors’ archive).

The microbial composition of kefir grains varies according to the region of origin and the substrate used for the proliferation of grains and the techniques used in their manufacture [24,25,26,27,28,29,30]. The main microbial genera found in kefir grains and beverage are shown in Figure 4. There are species from various genera of bacteria and yeasts [24,25,26,27,28,29,30,38,39].

Tavares et al. [39] evaluated kefir microbial diversity during the fermentation process by PCR–DGGE (Polymerase chain reaction denaturing gradient gel electrophoresis) molecular method and microbial species-level sequencing. Microbial diversity was distributed in four bacterial genera and four yeast genera. Bacteria from the genera *Lactobacillus, Lacticaseibacillus, Lentilactobacillus, Leuconostoc,* and *Acetobacter* were found, along with the yeasts *Saccharomyces, Kluvyeromyces, Lachancea*, and *Kazachstania*.

**Figure 5:**
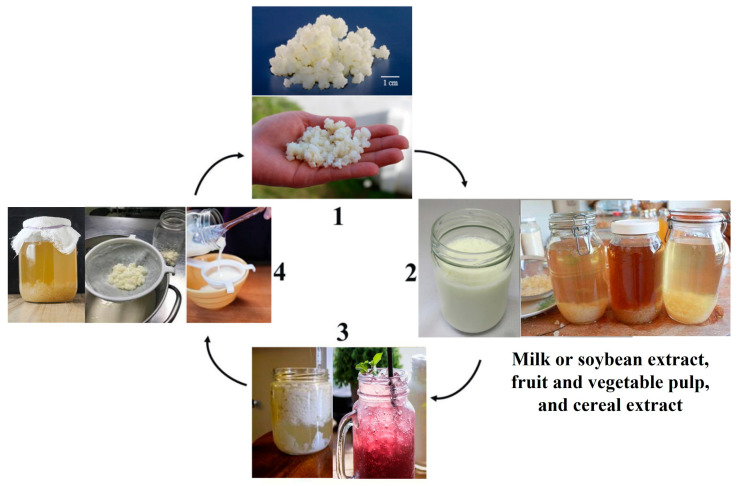
Production of kefir beverages. Kefir grains (1) are added to substratum and left at room temperature for fermentation for 18–24 h (2), the substratum is then fermented forming the kefir beverage (3), after which they are filtered, (4) and ready to start another cycle. The fermented beverage that results from step 3 is appropriate for consumption. Original and unpublished figure (authors’ archive).

Kefir grains are capable of fermenting on various substrates, such as cow, goat, sheep, and buffalo milk, brown sugar, fruit juices, soy extract, vegetables, and cereals [27,28,29,38,39]. The microbial species in kefir grains undergo three types of fermentation during the process: lactic, alcoholic, and acetic. This aspect, along with the adaptability of the grains, as described above, reflects the potential for new formulations and beverages [27,28,29,38,39]. The “traditional” way to produce kefir beverage is to use pasteurized or UHT (ultra-high-temperature processing) treated milk or brown sugar solution. However, other substrates can be used in the kefir grain fermentation process, such as soy/cereal extracts and fruit/vegetable pulp (Figure 5). After fermentation for 24–48 h, kefir beverage is ready to be consumed [27,28,29,38,39] (Figure 5).

### 4.3. Kombucha: Microbiome and Preparation

**Figure 6:**
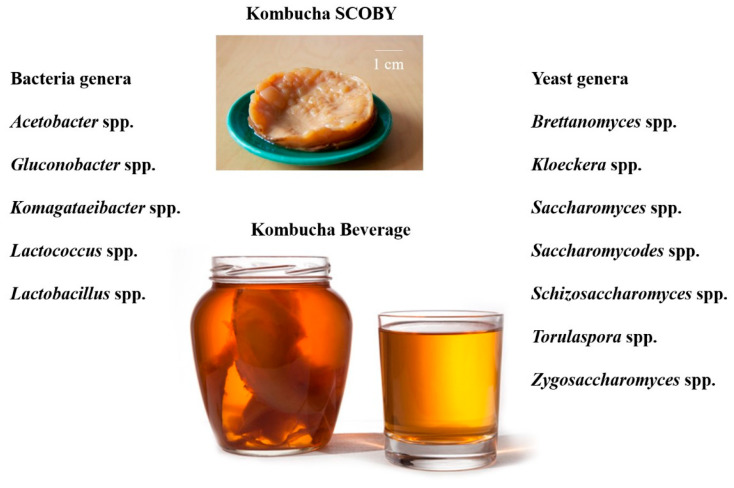
Bacteria/yeast genera present in kombucha SCOBY and kombucha-based beverage. Original and unpublished figure (authors’ archive).

Kombucha is a fermented beverage produced by the fermentation of black, oolong or green tea, varieties of *Camellia sinensis* tea, with sugar and a symbiotic microorganism culture known as SCOBY (Symbiotic Culture of Bacteria and Yeast) (Figure 6).

Many microorganisms are isolated from SCOBY, including acetic acid bacteria *(Acetobacter* spp., *Gluconobacter* spp. and *Komagataeibacter* spp.), lactic acid bacteria (*Lactococcus* spp. and *Lactobacillus* spp.) and yeasts (*Brettanomyces* spp., *Kloeckera* spp., *Saccharomyces* spp., *Saccharomycodes* spp., *Schizosaccharomyces* spp., *Torulaspora* spp., and *Zygosaccharomyces* spp.). The diversity of microorganisms with probiotic potential interacting with each other and with liquid environment is the reason for the beneficial properties of the beverage produced [40,41,42,43,44].

**Figure 7:**
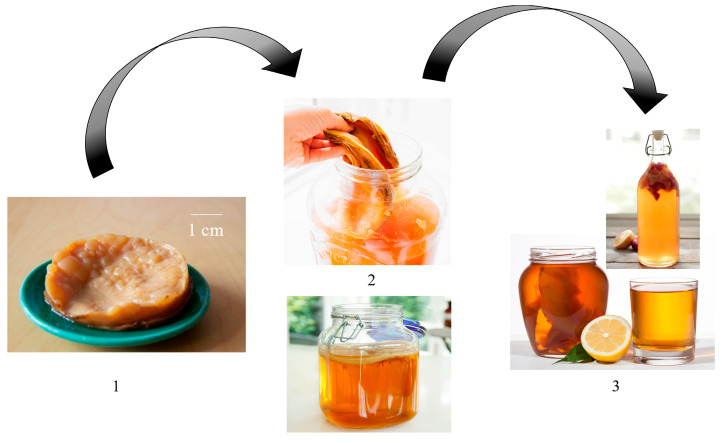
Kombucha production. SCOBY (1) are added to *Camelia sinensis* tea and are left to stand at room temperature for fermentation 168 h (2), after which the beverage can be re-fermented in another substrate (fruit, vegetable pulps) or be consumed (3). Original and unpublished figure (authors’ archive).

This kombucha beverage is fermented for a period of 7–12 days at room temperature in static fermentation (Figure 7). The organoleptic characteristics of the final beverage are modified based on the production environment, mainly temperature and the fermentation time. It is also important to add that the microorganisms from that beverage are distributed in two categories: those which are part of the cellulose biofilm and others which are struggling in the liquid [33,34].

### 4.4. Functional Properties of Kefir: Immunomodulatory, Antitumor/Anticarcinogenic, and Antimicrobial Activity

#### 4.4.1. Kefir Probiotic Microorganisms in Immunomodulatory Activity

Studies on the action of probiotic microorganisms on the host immune system have been carried out for some time [1,2,3,4,5,6,7,8,9,10,11,12,13,14,27,28,29,30,31]. Recent and non-recent bibliographies, most reporting studies with lactic acid bacteria, have shown that probiotics have an immunostimulant effect in animals and humans, although the mechanisms by which this occurs are not yet fully understood [1,2,3,4,5,6,7,8,9,10,11,12,13,14].

The immunostimulant effect may be related to the probiotic microorganism’s ability to interact with Peyer plaques and intestinal epithelial cells, stimulating IgA-producing B cells and migration of T cells from the gut [10]. The regular use of probiotics increases the macrophage phagocytic activity, stimulating the immune system [10].

Different strains of *Lactobacillus,* such as *Lactobacilli*, can be used in immunotherapeutic applications like oral vaccination therapy to induce T cell tolerance against autoimmune diseases [8]. Oral administration of *Lactobacillus reuteri* and *Lactobacillus brevis* has been shown to induce the expression of pro-inflammatory cytokines [8]. Consumption of the selected *Lactobacillus* strain provides a strategy to influence cytokine expression and generate an immune response in the human body, improving the host immune system [8].

#### 4.4.2. Kefir Probiotic Microorganisms with Antitumor/Anticarcinogenic Activity

Studies have been conducted using experimental mice or in vitro indicating some success in tumor size reduction with consumption of kefir beverage and grains. In the first report, an investigation of the antitumor effects of a water-soluble polysaccharide isolated from kefir grains indicates that the polysaccharide was able to inhibit the growth of Ehrlich carcinoma compared to control mice [45,46,47]. Furukawa et al. The authors of [48] indicated that feeding with kefir grain (2 g/kg body weight) was more effective in tumor inhibition (Lewis lung carcinoma) when administered 9 days after tumor inoculation in mice. Another study focused on the effects of oral administration of cow’s milk and kefir beverages (soy and milk) on tumor growth in mice inoculated with sarcoma-180 tumor cells resulting in ~65% and ~71% tumor growth inhibition, respectively, compared to controls [47].

Reis et al. [46] evaluated the effect of regular consumption of milk kefir beverage on the development of pre-neoplastic colonic lesions. Thirty Wistar rats were given water (Control group) or milk (Milk group) or kefir beverage (Kefir group) for five weeks. After that, colonic lesions were chemically induced, and the treatments continued for an additional thirteen weeks. Regular consumption of kefir beverage was able to reduce the incidence of aberrant crypt foci by 36%. The consumption of kefir beverage also increased the cecal concentration of short chain fatty acids, reduced the lactulose/mannitol ratio, and promoted an increase in the colonic concentration of TNF-α and IL-1β, and the enzyme catalase in comparison with the control group. Thus, milk kefir beverage reduced the development of lesions, probably by increasing the production of short chain fatty acids, with reduction of intestinal permeability, immunomodulation and improvement of colonic antioxidant activity.

#### 4.4.3. Kefir Probiotic Microorganisms with Antimicrobial Activity

The main probiotic effect of kefir is its ability to modulate the host gut microbiota, where there is a reduction in undesirable microorganisms and an increase in the number of beneficial microorganisms, such as *Lactobacillus* spp. and *Bifidobacterium* spp. [12,14,39,49,50,51]. Antimicrobial substances in kefir, such as lactic acid, acetic acid and H_2_O_2_, have been reported to have bactericidal and bacteriostatic effects on bacterial pathogens [12,14,39,49,50,51]. Lactic acid reduces the pH of the gastrointestinal tract, which is not suitable for the growth of pathogens [12,14,39,49,50,51]. In addition, H_2_O_2_ has an antagonistic and acetic acid has an antibacterial effect on pathogens [50]. *Salmonella* spp., *Yersinia* spp., *Shigella* spp., *Micrococcus* spp., *Escherichia coli, Bacillus cereus*, *Candida albicans* and *Klebsiella pneumoniae* are some of the pathogenic microorganisms inhibited by kefir [50]. In research that focused on the undesirable microbial inhibition activity of kefir, *Staphylococcus aureus*, *Pseudomonas fluorescens*, *Escherichia coli* and *Bacillus subtilis* were used as the target microorganisms, which are known to promote gastrointestinal diseases such as diarrhea. According to the results of the research, kefir showed microbial inhibition activity against all the target microorganisms [52].

There are many other studies that show the antibacterial activity of kefir and kefir grains against various pathogen bacteria and yeasts such as *Salmonella* spp., *Shigella* spp., *Staphylococcus* spp., *Pseudomonas* spp., *Bacillus* spp., *Streptococcus* pp., *Yersinia* spp. and *Micrococcus* spp. [53,54,55]. Kefir and its insoluble polysaccharide, kefiran, were both tested for antimicrobial activity against different bacterial species using an agar diffusion method. Both kefir and kefiran showed some activity against all tested pathogenic microorganisms; the highest activity was against *Streptococcus pyogenes* [53,54,55].

### 4.5. Functional Properties of Kombucha: Immunomodulatory, Antitumor/Anticarcinogenic, and Antimicrobial Activity

#### 4.5.1. Kombucha Probiotic with Immunomodulatory Activity

There are many recent studies on kombucha and its immunomodulatory activity. Zubaidah et al. [56] compared black tea kombucha and *Arabica* coffee leaves tea kombucha in terms of their immunomodulatory activity in mice infected with *Salmonella typhi*. They found that black tea kombucha was suitable for use as an immunomodulatory agent in mice infected with *S. typhi*. This result must be related to the composition of phenolic compounds and organic acids present in the beverage.

Sknepnek et al. [57] examined the possibility of using medicinal mushrooms for the development of new kombucha products, to increase their consumption. The products created were mushrooms’ fruiting bodies and 10% (*v/v*) actively fermenting black tea (*Camellia sinensis* L.) kombucha broth. Sknepnek et al. [57] also analyzed the biological properties of kombucha polysaccharides, finding highly desirable immunomodulatory properties in human cell cultures. The results obtained regarding the inhibitory effects of kombucha extracts on Th2 and IL-10 suggest that the consumption of kombucha beverages prepared on edible mushroom substrates, as well as the consumption of isolated polysaccharide extracts, may be beneficial for the prevention and treatment of Th2-mediated immunopathology, such as allergic reactions, asthma or atopic dermatitis. In addition, the stimulation of pro-inflammatory cytokines and reduction of anti-inflammatory IL-10 cytokine after treatment of PHA-stimulated PBMCs with kombucha mushroom polysaccharide extracts may contribute to the body’s defense potential against external pathogens, such as viruses. Further studies are needed to confirm these findings; however, the results suggest that the consumption of kombucha may hold promise for improving the immune response of the human body.

In another study, the objective was to investigate the antioxidant activity and anti-inflammatory effects of Kombucha produced with oak (KAO), by examining its ability to modulate in TNF-alpha and IL-6 derived from macrophages. Among the results, the levels of pro-inflammatory cytokines IL-6 and TNF-alpha were significantly reduced by the sample treatment. Likewise, the nitric oxide (NO) production was lower in the treatment with kombucha, and KAO compared to LPS-stimulated macrophages. Fermented oak beverages negatively regulated NO production, while pro-inflammatory cytokines (TNF-alpha and IL-6) in macrophages were stimulated with LPS. In addition, the phytochemicals present in KAO decrease oxidative stress [58].

#### 4.5.2. Kombucha Probiotic with Antitumor/Anticarcinogenic Activity

There are several studies on the anticarcinogenic potential of kombucha [59,60,61]. Ziska et al. [59] investigated the cytotoxic activity of *Solanum nigrum* L. n-hexane fruit extract against MCF-7 breast cancer cell line. In this study, two fermentations were carried out with kombucha and then 20% (*v/v*) of the previous beverage was added as inoculum for fermentation of the extract, with addition of 1.5% (*w/v*) n-hexane fruit extract, and 20% (*w/v*) white sugar. Among the interesting results found in this study, flavonoids and steroids were identified from the extract and GC–MS. However, the cytotoxic activity did not show significant results for the MCF-7 breast cancer cell line.

In contrast, promising results were found in a study conducted by Ghodousi Dehnavi et al. [60] which evaluated the effect of different fractions of Kombucha tea on the proliferation and apoptosis of the HT-29 colon cancer strain, yielding promising results. The findings revealed that the cell growth inhibition was dose dependent. The IC50 concentration in these fractions caused DNA fragmentation. In this study, cell death occurred via the apoptotic pathway and showed anti-proliferative activity. This demonstrated that kombucha can be a good candidate for inhibiting the proliferation of colorectal cancer cells.

Another study aimed to investigate the cytotoxicity of kombucha obtained from samples of different teas against the colorectal cancer cell line and found promising results. Kombucha tea was prepared with 1% green tea, oolong tea or black tea and 10% sucrose with the initial culture of kombucha tea: a consortium culture combining yeast and acetic acid bacteria. Kombucha prepared from green tea and black tea demonstrated toxicity in colorectal cancer cells Caco-2 [61].

#### 4.5.3. Kombucha Probiotic with Antimicrobial Activity

The antimicrobial properties of kombucha have been studied in different conditions and for different applications, from food to environmental settings. Kombucha production is a homemade culture, so the kombucha environment is not sterile. However, over the years, several researchers studying different kombucha colonies have not found a high contamination of the colonies with other microorganisms to be pathogenic. In addition to the symbiotic/probiotic culture having antimicrobial potential, the high acidity and alcohol content influence the absence of contaminants [62].

In a recent study, Laurenson et al. [63] tested the effectiveness of Kombucha SCOBY (symbiotic culture of bacteria and yeast) in reducing the concentration of *Escherichia coli* in dairy effluent shed (SDR). In this study, the SCOBY kombucha was highly effective in reducing the number of *E. coli* colony forming units (CFU) to undetectable levels. The decrease in CFUs occurred within 48 h after inoculation of the Kombucha SCOBY into the effluent matrix, and there was also a significant decrease in the pH of this effluent.

Santos Júnior et al. [64], seeking to analyze the consumption of fermented kombucha conducted in a Brazilian hospital, provided important results on the beverage’s antimicrobial activity. The fermented growth was efficient against *Microsporum canis* (LM-828), *Escherichia coli* (CCT-0355) and *Salmonella typhi* (CCT-1511). The best inhibition conditions against *M. canis* (>32 mm) and *E. coli* (16 mm) were observed at pH 4.0, 55% commercial sugar and 0.10 g/L MgSO_4_, and for *S. typhi* (32 mm) without MgSO_4_.

Battikh et al. [65], aiming to compare the antimicrobial activities of two kombucha beverages fermented from green and black teas and to characterize the antimicrobial compounds, found interesting results for antifungal and antibacterial response. The results showed interesting antimicrobial potentials of both kombucha teas tested against the tested microorganisms, except *Candida krusei*. Fermented green tea exhibited the highest antimicrobial potential, against *Staphylococcus epidermidis*, *Listeria monocytogenes* and *Micrococcus luteus*. In addition, an interesting anti-*Candida* potential was revealed by the reaction of green tea kombucha against *Candida parapsilosis.*

Another study by Kaewkod et al. [61] investigates the antioxidant and antibacterial properties of kombucha obtained from different types of *C. sinensis*, including green tea, oolong tea, and black tea. They found that pathogenic enteric bacteria: *Escherichia coli. E. coli* O157: H7, *Shigella dysenteriae*, *Salmonella typhi*, and *Vibrio cholera* were inhibited by kombucha.

### 4.6. Technological Advances for Improving Kefir and Kombucha Functional Properties

Atalar and Dervisoglu [66] utilized a methodology based on the optimization of the parameters of the spray drying process of kefir powder using response surface methodology (RSM). This study proposes an alternative production of kefir. This could bring benefits for those seeking simple and quick consumption at home without having to grow the grains. The optimum spray drying conditions were combined with the lyophilization results. The findings showed that, at the ideal point, high quality kefir could be obtained as lyophilized powder. Teijeiro et al. [67] also evaluated the suitability of producing kefir powder using spray drying. The authors assessed the survival of microorganisms after drying, storage and simulated gastrointestinal conditions. The results demonstrated that spray drying of kefir is an appropriate approach to obtaining a concentrated and efficient product. Spray drying of kefir leads to many advantages for storage and transportation, in addition to containing viable and active microorganisms.

Yilmaz et al. [68] studied an alternative way of encapsulating probiotics within alginate nanofibers by electrospinning, monitored under simulated gastrointestinal conditions. Probiotic viability was determined under simulated gastrointestinal and in vitro conditions. Nanoencapsulation of the *Lactobacillus paracasei* KS-199 strain isolated from kefir could increase its survival in simulated gastric juice and improved its viability/survival in the gut. Given the results, it was possible to suggest that alginate could be an important biopolymer playing an essential role in probiotics encapsulation for greater viability. Nano-encapsulated *Lactobacillus paracasei* KS-199 strain demonstrated higher survival during its passage through the stomach and colonization of the gut.

Jayabalan et al. [69] studied heat treatments to test the long-term storage of kombucha and prevent biofilm formation in the final product. The study revealed the influence of heat on the biochemical constituents and free radical scavenging properties of kombucha tea. The heat treatment at 60, 65 and 68 °C for 1 min controlled biofilm formation in kombucha tea without changing its clarity and flavor. However, polyphenols in the tea and the quality parameters of black tea varied during the storage period. It was concluded that heat treatment was not an adequate method for preserving kombucha tea.

Roby et al. [70] carried out a study with the objective of producing fermented dough bread using an encapsulated kombucha starter culture without the addition of baker’s yeast. Principal component analyzes showed the presence of 15 metabolites in the kombucha fermented dough. The main compounds contributing to differences in sourdough yeast without kombucha were alpha-aminobutyric acid, alanine, acetic acid, riboflavin, pyridoxine, anserine, tryptophan, gluconic acid and trehalose. The encapsulated kombucha yeast starter increased the volume of the bread compared to the yeast bread. The kombucha as an encapsulated fermented dough starter extended the life of the bread by 5 to 10 days at room temperature. Dough bread prepared using the encapsulated kombucha dough initiator showed higher flavor scores and overall acceptability compared to the other bread. The findings indicate that the encapsulated kombucha yeast starter promises to produce functional yeast dough bread with extended shelf life and improved quality.

### 4.7. An Informed Opinion and Manuscript Limitation

Kefir and kombucha are functional foods with several therapeutic benefits, as described throughout this review: Kefir and Kombucha possesses anti-inflammatory activity, antibacterial activity, anticarcinogenic potential, antimicrobial activity, antioxidant activity, activities against mental disorders and anti-proliferative activity, which have currently been studied. However, most of the health benefits are not scientifically proven in human clinical models. Future studies hold promise in scientifically demonstrating the claimed health benefits.

Despite the benefits, there are some limitations to kefir and kombucha consumption. Consumption of these cultures can be harmful only if they are incorrectly prepared and if individuals with preexisting conditions prove intolerant to the characteristics of the fermentative process of kefir and kombucha. As these cultures contain acetic acid and lactic acid, they possess an acidic pH, which can be limiting for some individuals with stomach disorders. However, research on the stomach, skin, lactose intolerance, hyperlipidemia, and sensorimotor behavior is continuously increasing.

Regarding safety for consumption, the main reasons for culture contamination include unreliable raw materials, vessels, packages, and a lack of sanitation during the fermentation process. This can lead to exposure to undesirable metabolites and microorganisms. Therefore, some protocols and standards should be framed by health organizations to standardize production methods, raw materials, and quality control. At the same time, limitations regarding this study are also noted regarding the databases and languages Other scientific databases can be used worldwide by students, teachers/researchers, and professionals. However, the databases investigated in this study cover the available global literature. In relation to the three languages (English, Portuguese and Spanish), there is a limitation in relation to different countries. The authors chose English, Portuguese and Spanish, as they are used in their countries of residence. However, the English language is considered universal in terms of globally reaching scientific publications.

## 5. Conclusions and Prospects

The boxplots analysis revealed a summary of a dataset “Index Terms—Keywords” for kefir and kombucha. Google Scholar was the database with the highest number of articles found, when the search for the keywords used in the study (containing ~4 × 10^6^–~4 million articles available). Following Google Scholar was the Science Direct database, containing ~3 × 10^6^–~3 million articles available, and the BVS databases—Biblioteca Virtual de Saúde (Virtual Health Library) and Lilacs, both containing a value of ~2 × 10^6^–~2 million articles available. The databases containing the lowest number of articles found were Nutrients and Medline, both with a value of ≤0.1 × 10^6^–≤100 thousand articles.

Kefir and kombucha have been known as complex probiotic cultures for centuries, as they have unique chemical and microbiological compositions. Many scientific studies indicate that kefir and kombucha certainly contain various functional properties, such as antimicrobial, antitumor, anticarcinogenic and immunomodulatory activity, in addition to having a microbiological composition of probiotic bacteria and yeasts. These fermented probiotic cultures have great probiotic/functional potential of scientific interest. Table 3 summarizes the main microbiological/probiotic/functional characteristics when comparing kefir and kombucha.

Further laboratory and clinical studies should be carried out on kefir and kombucha, to analyze other therapeutic and functional properties that have not been noticed. Fermented cultures/beverages such as kombucha and kefir are key opportunities for the food and clinic/medical fields.

## Figures and Tables

**Table 1:** Quantitative analysis of scientific articles until January 2024 in the Database Google Scholar.

Languages	Key Words	Number of Articles
English	probiotics microorganisms	993,000
	kefir probiotic	779,000
	kombucha probiotic	707,000
	non-dairy probiotic beverage	747,600
	functional beverage	509,000
	functional kefir beverage	457,600
	functional kombucha beverage	342,500
	symbiotic culture	132,300
	kefiran	7321
Spanish	microorganismos probióticos	416,500
	kéfir probiótico	365,000
	probiótico kombucha	178,988
	bebida probiótica no láctea	116,900
	bebida funcional	109,000
	bebida de kéfir funcional	93,800
	bebida kombucha funcional	63,702
	cultivo simbiótico	42,810
	kefiran	3000
Portuguese	micro-organismos probióticos	116,800
	kefir probiótico	93,510
	kombucha probiótico	47,751
	bebida probiótica não láctea	33,200
	bebida funcional	27,200
	bebida funcional de kefir	15,500
	bebida funcional de kombucha	1292
	cultura simbiótica	1270
	kefiran	1300

**Table 2:** Quantitative analysis of scientific articles until January 2024 in the Database Science Direct.

Languages	Key Words	Number of Articles
English	probiotics microorganisms	19,184
	kefir probiotic	2168
	kombucha probiotic	564
	non-dairy probiotic beverage	3570
	functional beverage	6923
	functional kefir beverage	2517
	functional kombucha beverage	459
	symbiotic culture	31,111
	kefiran	538
Spanish	microorganismos probióticos	272
	kéfir probiótico	13
	probiótico kombucha	39
	bebida probiótica no láctea	0
	bebida funcional	921
	bebida de kéfir funcional	0
	bebida kombucha funcional	0
	cultivo simbiótico	33
	kefiran	23
Portuguese	micro-organismos probióticos	0
	kefir probiótico	0
	kombucha probiótico	0
	bebida probiótica não láctea	0
	bebida funcional	0
	bebida funcional de kefir	0
	bebida funcional de kombucha	0
	cultura simbiótica	0
	kefiran	0

**Table 3:** Main microbiological/probiotic/functional characteristics when comparing kefir and kombucha.

	Kefir	Kombucha
Origins	Northern Caucasian mountains [49,70,71,72].	East Asia [69,73,74].
Principle characteristics	Kefir is a fermented beverage and effervescent. The fermentation process is conducted by the kefiran polysaccharide matrix [6,7,13,15,16,17,18,19,20,21,22,23,24,25,26,27,28,29,30,31,39,45,46,47,48,49,50,52,53,54,55,66,67,68,72,75,76,77,78,79,80,81].	Kombucha is a fermented beverage produced by the fermentation of black, oolong or green tea, varieties of *Camellia sinensis* tea, with sugar and a symbiotic microorganism culture known as SCOBY (Symbiotic Culture of Bacteria and Yeast) [5,13,32,33,34,35,40,41,42,43,44,56,57,58,59,60,61,62,63,64,65,69,70,71,73,76,77,78,79,80,82,83].
Probiotic microbioma	*Lactobacillus* spp., *Acetobacter* spp., *Bifidobacterium* spp., and others; and yeast species as *Saccharomyces* spp., *Candida* spp., *Kluyveromyces* spp., [6,7,13,15,16,17,18,19,20,21,22,23,24,25,26,27,28,29,30,31,39,45,46,47,48,49,50,52,53,54,55,66,67,68,72].	*Acetobacter* spp., *Gluconobacter* spp., *Komagataeibacter* spp., *Lactococcus* spp., *Lactobacillus* spp., *Brettanomyces* spp., *Kloeckera* spp., *Saccharomyces* spp., *Saccharomycodes* spp., *Schizosaccharomyces* spp., *Torulaspora* spp., and *Zygosaccharomyces* spp. [5,13,32,33,34,35,40,41,42,43,44,56,57,58,59,60,61,62,63,64,65,69,70,73].
Diet-kefir/kombucha against Obesity	Kefir on adiposity and gut microbiota [77,78,79].	Kombucha on adiposity and gut microbiota [77,78,79].
Tradicional preparation of the beverages	Kefir beverage is prepared using pasteurized or UHT (Ultra-high-temperature processing) treated milk and the fermentation occurs for 24–48 h [27].	Kombucha beverage is fermented for a period of 7–12 days at room temperature in static fermentation [74].
Immunomodulatory activity	Kombucha tea/beverage was used as an immunomodulating agent in mice infected with *Salmonella typhi* [56]. This showed inhibitory effects on Th2 and IL-10 suggesting that the consumption of these beverages may be beneficial for the prevention and treatment of allergic reactions, asthma, or atopic dermatitis [57].	Kefir increases the phagocytic activity of macrophages, stimulating the immune system. Kefir also influences the activity of host immune cells, regulation of inflammation, barrier function and cell-to-cell signaling [10].
Antitumor/anticarcinogenic activity	Feeding with kefir grain was effective in inhibiting lung carcinoma when administered for 9 days after tumor inoculation in mice [48]. Another study demonstrated that kefir beverages (soybeans and milk) resulted in ~65% and ~71% inhibition of tumor growth (sarcoma-180) compared to controls [47] The gut microbiota influenced by kefir probiotic intake may support well-being and alleviate intestinal inflammation and colon cancer [80].	In a study with the HT-29 colon cancer strain, kombucha was capable of inhibition of cell growth, DNA fragmentation, cell death apoptosis and antiproliferative activity [60]. Kombucha prepared from green tea and black tea has shown toxicity in Caco-2 colorectal cancer cells [61]. The gut microbiota influenced by kombucha probiotic intake may support well-being and alleviate intestinal inflammation and colon cancer [80].
Antimicrobial activity	Kefir contains antimicrobial substances, such as lactic acid, acetic acid and H_2_O_2_, which have bactericidal and bacteriostatic effects [75]. Kefir showed microbial inhibition activity against *Staphylococcus aureus, Pseudomonas fluorescens, Escherichia coli* and *Bacillus subtilis* [52].	Kombucha was highly effective in reducing the number of *Eschericha coli* colony-forming units (CFU) to undetectable levels [63]. Pathogenic bacteria: *Escherichia coli* O157:H7, *Shigella dysenteriae, Salmonella Typhi* and *Vibrio cholera* were inhibited by kombucha [61].
Manipulation and new formulations	The production of kefir powder using spray drying helps the survival of kefir probiotics microorganisms in the gastrointestinal environment [66,67].	The sustainable microbial biopolymer kombucha shows the potential to be used as a bioink for 3D bioprinting [71]. Production of fermented dough bread uses a starter culture of encapsulated kombucha without addition of baker’s yeast [70].

## Data Availability

Data available on request.
